# Bilateral Central Retinal Vein Occlusion, multiple dental implants and severe glomerulonephtitis – Any connection?

**Published:** 2019

**Authors:** Monica Malaescu, Bogdana Tabacaru, Simona Stanca, Tudor Horia Stanca

**Affiliations:** * “Prof. Dr. Agrippa Ionescu” Emergency Clinical Hospital, Bucharest, Romania; ** “Carol Davila” University of Medicine and Pharmacy, Bucharest, Romania; *** Pediatric Clinic, “Grigore Alexandrescu” Children Emergency Hospital, Bucharest, Romania

**Keywords:** Central Retinal Vein Occlusion, bilateral, chronic macular edema, cotton wool spots, glomerulonephritis, dental implants

## Abstract

We present a case of Bilateral Central Retinal Vein Occlusion in a patient who received 11 dental implants and later developed idiopathic glomerulonephritis with renal failure.

## Introduction

Retinal vein occlusion is the most frequent primary vascular disorder of the retina. Central retinal vein occlusion occurs at all ages, with a mean age between 60 and 70 years. It usually involves one eye, but 5 to 11% of the patients will suffer from occlusion in the other eye within five years [**1**-**3**].

Symptoms of central retinal vein occlusion include blurred vision, floaters, black spots, or metamorphopsia. The blurred vision may involve the eye after getting up in the morning, and fade during the day caused by the change in venous return. The visual acuity might deteriorate over a couple of days, leading the patient to visit the ophthalmologist only after 1-3 weeks. The patient usually presents with a visual acuity of 20/ 200 - 20/ 40 [**1**,**4**].

Risk factors for CRVO are cardiovascular, local (glaucoma, retinal vasculitis, optic disk drusen, and trauma), coagulation disorders, and hyperviscosity syndromes [**5**].

The fundus takes various aspects depending on the time passed from the occlusion. Early features range from superficial flame shaped hemorrhages, engorged veins, a swelled, hyperemic disk, to dense hemorrhages that include more deeply located dot and blot bleedings, dilated tortuous veins, and important papilledema. Local ischemia can be observed as cotton wool spots. Macular edema is always present. Late features include resorption of the hemorrhages beginning with the superficial ones, vein caliber reduces but vessel walls lose their transparency and gain a whitened aspect, and a macular scar can remain after the reduction of the edema [**1**].

## Case report

A 54-year-old patient was admitted in our clinic in 2013 for sudden decrease of visual acuity in both eyes shortly after he received 11 dental implants, performed over a period of 1 year. General symptoms were absent at admission.

The patient had no relevant family history or ophthalmological afflictions, but he was known with untreated chronic hepatitis C diagnosed in 2005 and received 11 dental implants in the previous year.

At presentation, his best-corrected visual acuity was 20/ 100 (0.7 logMAR) for the right eye and 20/ 200 (1 logMAR) for the left eye with a small spherical hyperopic correction. The intraocular pressure by applanation tonometry was 15 mmHg in the right eye and 17 mmHg in the left eye.

The findings on external examination and slit-lamp examination of the anterior segment were within normal limits.

The fundus of each eye was examined after pharmaceutical mydriasis with 0.5% tropicamide and 10% phenylephrine hydrochloride ophthalmic solutions. There was a typical “blood and thunder fundus” appearance with many retinal splinter and spot hemorrhages and cotton wool spots in all quadrants. The retinal arteries were narrowed, the veins were turgescent, and the vessels had a concentric arrangement. The optic nerve disc in both eyes was imprecisely delimited, had a swollen appearance and the cupping was absent, this aspect being highly suggestive for papillary edema. There was no fundus camera photography taken at presentation. 

**Fig. 1:**
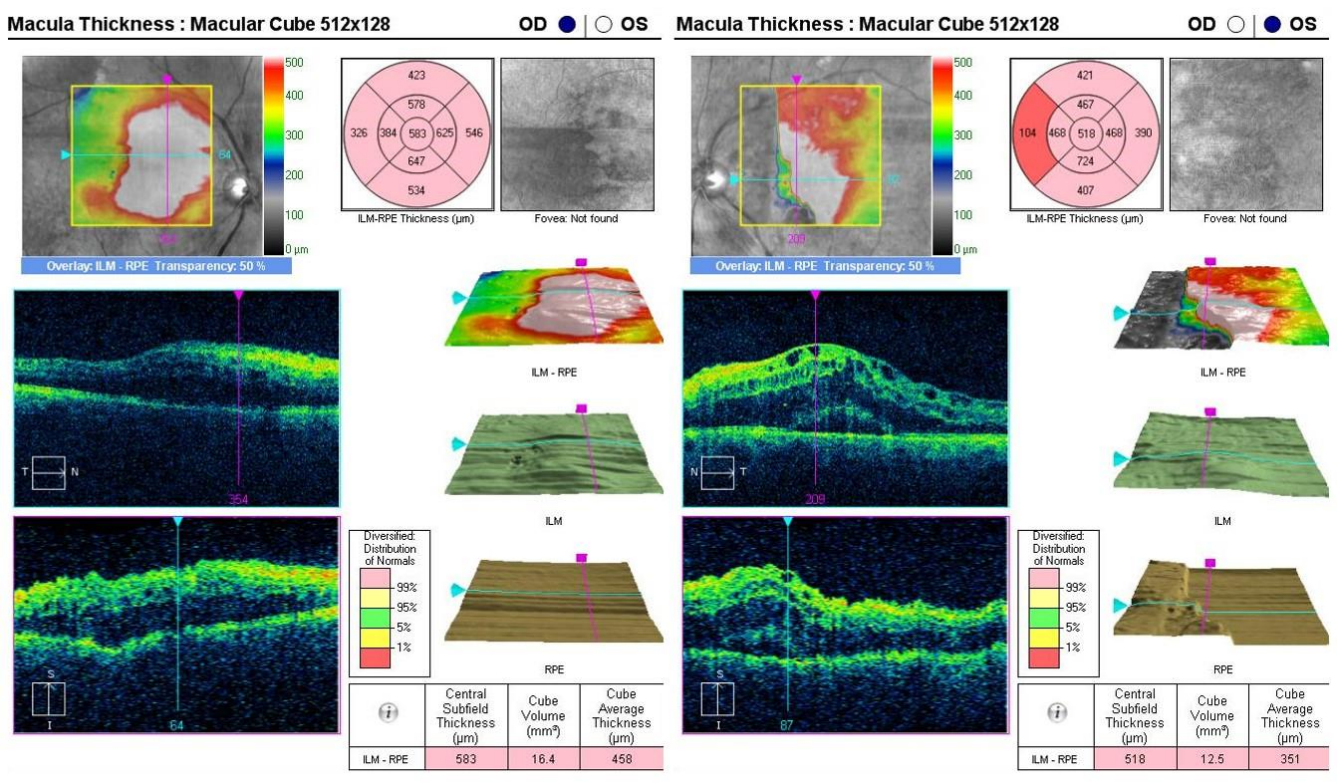
Optical coherence tomography shows thickening of the retinal layers in the macular region, diffuse intraretinal fluid in the right eye, with cystic spaces and subretinal fluid in the left eye

The macular cube optical coherence tomography analysis revealed thickening of the retinal layers, diffuse intraretinal fluid in the right eye and cystoid spaces, and subretinal fluid (neuro sensorial layer decollation) in the left eye (**Fig. 1**).

Based on this clinical and paraclinical investigations we established the working diagnosis of Bilateral Central Retinal Vein Occlusion with Non-tractional Macular Edema OS>OD. The patient was further investigated in order to establish the etiological diagnosis and the course of treatment. 

We further recommended a series of clinical, paraclinical and laboratory complementary investigations (**Table 1**). The complete blood count, coagulogram, and erythrocyte sedimentation rate had normal values; the biochemistry showed high AST, ALT, and GGT levels, caused by the hepatitis C infection and normal urea and creatinine values. Glucose levels were normal. There were no significant findings at the cardio-vascular and rheumatologic exam. The patient postponed the nephrological exam. 

**Table 1:** Recommended investigations and results

Recommendations	Results
Hemoleucogram	• Normal
Coagulogram	• Normal
Biochemistry	• ↑AST, ALT, GGT
	• N- Urea, Creatinine
Nephrological exam	• postpones exam
Blood pressure	• Normal
Glucose levels	• Normal
Cardio-vascular exam	• Normal
Rheumatologic exam	• ANA, PANCA, anti-DNA, anti-cardiolipin, anti- glomerular basement membrane antibodies – negative
	• Lupus anti-coagulant - absent
	• Cryoglobulins – absent

Considering these complementary investigations, we established the diagnosis of Bilateral Central Retinal Vein Occlusion with Non-tractional Macular Edema OS>OD.

The differential diagnosis included causes of papillary edema associated with retinal hemorrhages and cotton wool spots (**Table 2**). Diabetic retinopathy, hypertensive retinopathy, and infections were taken into consideration, but the patient had normal glucose levels, normal blood pressure, and normal complete blood count at the onset of the pathology.

**Table 2:** Differential diagnosis and exclusion factors

Differential diagnosis	Exclusion factors
Hypertensive retinopathy	Normal blood pressure
Diabetic retinopathy	Normal glucose levels
Ocular ischemia	Normal carotid Echo-Doppler
Hyper viscosity syndromes	Normal laboratory results
Inflammatory diseases	
	Normal inflammatory markers
Infections	Normal laboratory results

Given the above exclusion criteria and the fact that the patient presented with several elements common for Central Retinal Vein Occlusion, our positive diagnosis was confirmed.

The patient was followed-up for 1 year. He received vasodilator therapy (Nicergolin 30mg/ day), antithrombotic (Acetyl salicylic acid 75mg/ day), and neuroprotector treatment. He also received pro renata treatment with anti-VEGF and triamcinolone acetonide intravitreal injections in both eyes.

In the following years, the general condition of the patient deteriorated, with the development of several systemic pathologies. In 2014, he started statin treatment due to moderate dyslipidemia. He also developed pretibial edema and polyserositis but postponed a nephrological and cardiological examination. However, due to a hypertensive jump, he was admitted in a cardiology clinic where he was diagnosed with Arterial Hypertension nonresponsive to treatment, Hypertensive Cardiomyopathy and NYHA II Heart Failure. After the detection of high urea and creatinine levels, and nephrotic proteinuria, he was transferred to the nephrology clinic where he was diagnosed with Nephrotic Syndrome and Stage III Renal Failure. A renal biopsy revealed the aspect of Diabetic Glomerulonephritis despite normal glucose levels, leading to the diagnosis of Idiopathic Glomerulonephritis. He started prednisolone therapy but developed steroid induced diabetes and insulin therapy had to be initiated for a brief period, with the normalization of the glycemia after the stop of the steroid treatment. In 2015, the patient was diagnosed with chronic obstructive arteriopathy due to atherosclerosis after he developed septic syndrome from an infected gangrene of the right foot followed by right transtarsal foot amputation. A few months after the diagnosis, he develops left foot gangrene, followed by left transmetatarsal foot amputation. Meanwhile, a jeun glycemia started to rise and he had to start treatment with oral antidiabetics. The patient also began treatment with Exviera and Viekirax for the infection with C hepatic virus. Due to severe polyserositis, the patient began hemodialysis and the arterial hypertension resided shortly after, with no further treatment. In 2017, he received a renal transplant from a living related donor and started immunosuppression therapy. A year after, he suffered from bacterial endocarditis with MRSA and required minimally invasive mitral valve replacement.

After a four-year absence from the ophthalmologist, the patient returned in our clinic in 2018.

**Fig. 2:**
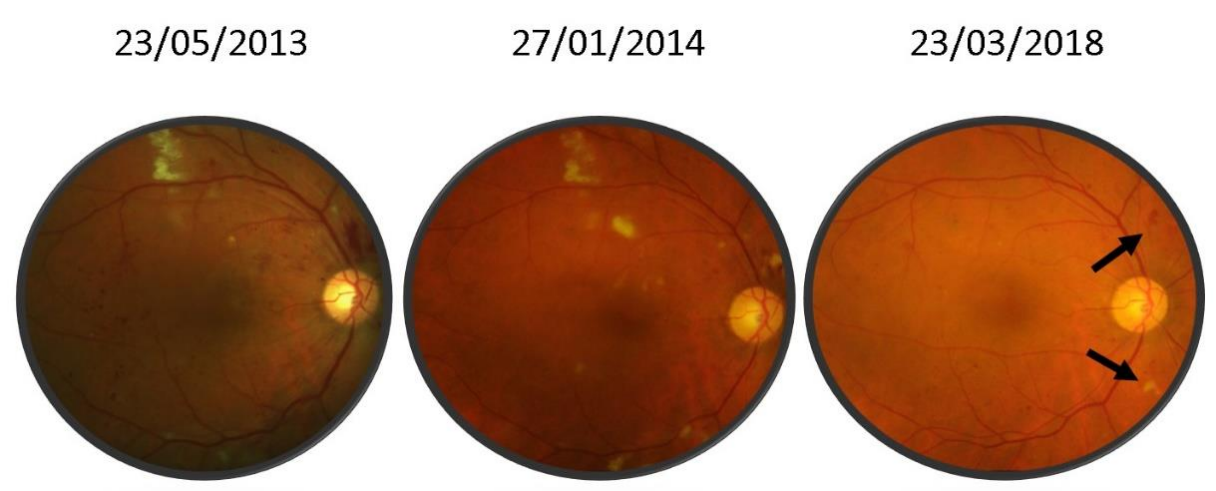
Color fundus photography of the right eye between 2013 and 2018. Persistent retinal hemorrhage and cotton wool spot (black arrows)

**Fig. 3:**
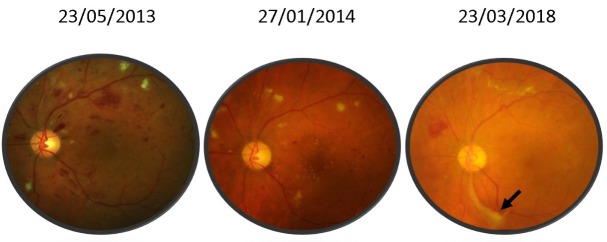
Color fundus photography of the left eye between 2013 and 2018. Fibrovascular membrane along the infero-temporal vascular arcade (black arrow)

**Fig. 4:**
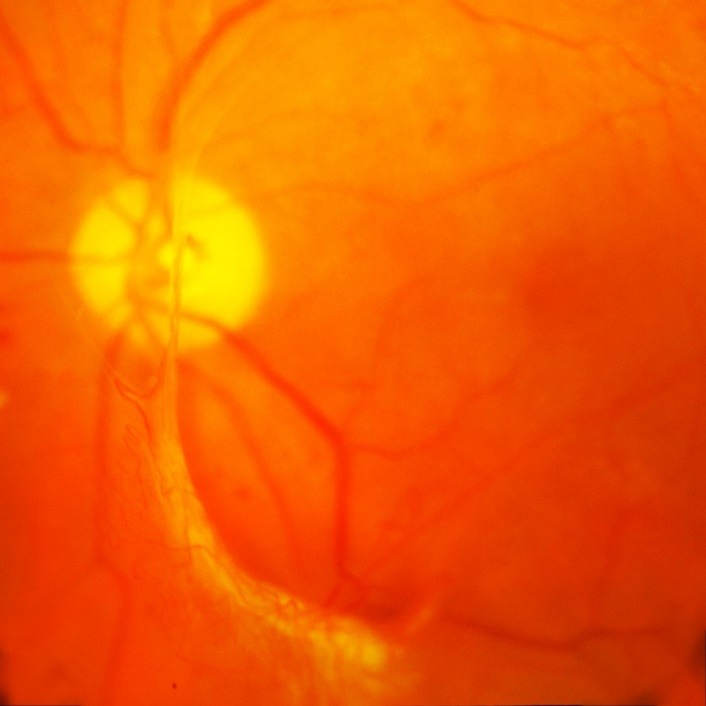
Fibro-vascular membrane – detail

For the left eye, best corrected central visual acuity increased for the right eye from 20/ 100 (0.7 logMAR) to 20/ 25 (0.1 logMAR) and for the left eye from 20/ 200 (1 logMAR) to 20/ 80 (0.6 logMAR). The aspect of the optic disc improved with the complete remission of the papillary edema. Retinal hemorrhages and cotton wool spots disappeared almost completely (**Fig. 2**,**3**), however the left eye developed a fibro-vascular membrane along the temporal inferior vascular arcade causing traction to the retina (**Fig. 4**).

**Fig. 5:**
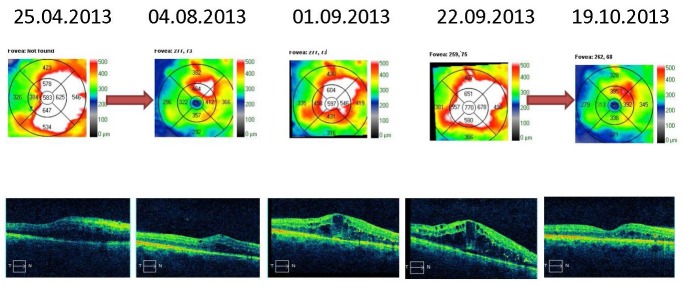
Macular thickness OCT – right eye - recurrent macular edema treated by PRN intravitreal injections with anti-VEGF and triamcinolone acetonide (red arrows)

**Fig. 6:**
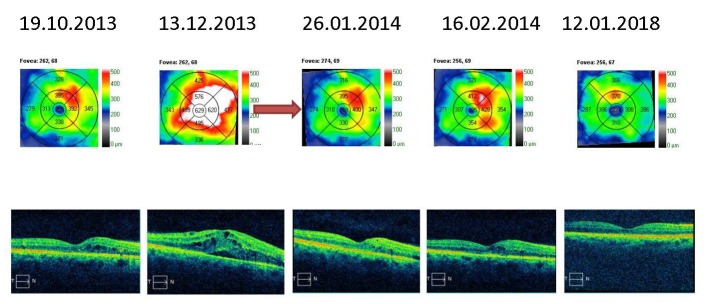
Macular thickness OCT – right eye - recurrent macular edema treated by PRN intravitreal injections with anti-VEGF and triamcinolone acetonide (red arrow)

**Fig. 7:**
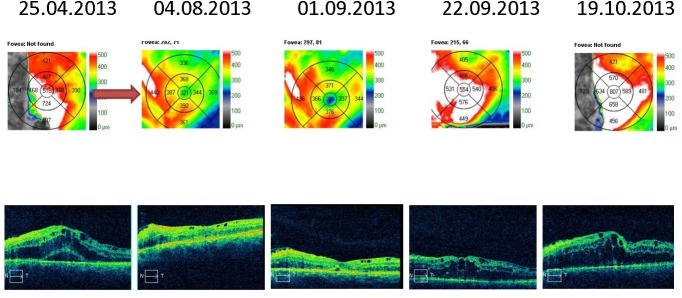
Macular thickness OCT – left eye - recurrent macular edema treated by PRN intravitreal injections with anti-VEGF and triamcinolone acetonide (red arrow)

**Fig. 8:**
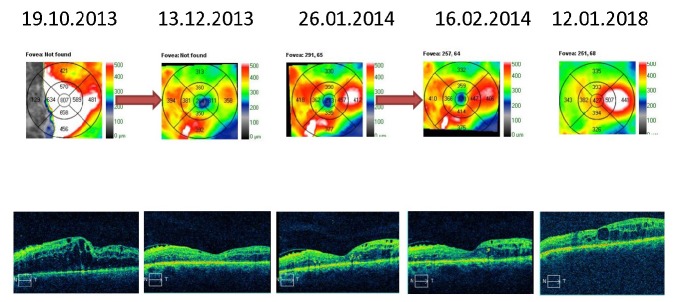
Macular thickness OCT – left eye - recurrent macular edema treated by PRN intravitreal injections with anti-VEGF and triamcinolone acetonide (red arrows)

The macular thickness OCT analysis varied in time. The right eye had recurrent macular edema episodes, treated by pro renata intravitreal injections with anti-VEGF and triamcinolone acetonide. The retinal thickness stabilized with almost complete remission of the macular edema (**Fig. 5**,**6**). The same observations could be made for the left eye; however, the edema was greater and the response to the intravitreal injections weaker. At the last examination, the patient still had intra-retinal fluid situated between the macula and the papilla (**Fig. 7**,**8**).

The patient received scatter pan-retinal photocoagulation in the left eye for the fibrovascular membrane and grid micro-pulse laser in both eyes for the remaining macular edema.

## Discussion

Particular for this case was the fact that the patient developed bilateral central retinal vein occlusion shortly after undergoing extensive dental interventions, with normal clinical and paraclinical values prior to this. A year after the incident, he developed glomerulonephritis of unknown etiology which lead to severe metabolic imbalance and a great number of complications.

Several risk factors were taken into consideration, in order to establish the mechanism that led to central retinal vein occlusion.

Cardiovascular risk factors (arterial hypertension, cardiovascular disease, diabetes mellitus, obesity, hyperlipemia, smoking) are associated with this type of retinal vascular disease [**1**,**6**-**11**]. However, the patient had normal BMI, had quit smoking 5 years prior to this event, and developed hyperlipemia, arterial hypertension, cardiovascular disease and diabetes mellitus a year after the CRVO, as complications of the renal failure, which led to a metabolic imbalance.

Hyperviscosity of the blood can appear under several circumstances. Hyperviscosity syndromes (leukemia, polycythemia vera, essential thrombocythemia, idiopathic myelofibrosis, macroglobulinemia, myeloma) [**1**] were excluded. Increased hematocrit, plasma viscosity [**1**,**12**-**14**] red cell aggregation [**15**], plasma fibrinogen [**16**] and reduced red cell deformability [**17**,**18**] were also excluded, with normal laboratory results prior and shortly after the occlusive event. 

High homocysteine levels are associated with thromboembolic events, by causing increased tissue factor expression, attenuated anticoagulant processes, enhanced platelet reactivity, increased thrombin generation, augmented factor V activity, impaired fibrinolytic potential, and vascular injury, including endothelial dysfunction [**19**]. Hyper-homocysteinemia testing was negative.

Thrombophilia, a hematological disorder associated with vascular occlusions, can be hereditary or acquired. Hereditary thrombophilia implies mutations on genes that cause decreased factor V Leiden, factor XII [**20**,**21**], antithrombin III [**18**,**20**], protein C or protein S, and activated protein C resistance (APCR) [**1**,**21**-**24**]. Acquired thrombophilia is identified by immunoserological testing for lupus anticoagulant, anticardiolipin, antiphospholipid and anti-β2-glycoprotein 1 antibodies [**25**-**27**]. Tests were negative for both hereditary and acquired thrombophilia. 

Nephrotic syndrome and glomerulonephritis had been associated with underlying hemostatic derangement in kidney disease patients, including activation of procoagulants, decreased production of endogenous anticoagulants, enhanced platelet activation and aggregation, platelet dysfunction and decreased fibrinolytic activity [**28**-**36**]. The Beijing study concluded that in a Northern Chinese population, chronic kidney disease as defined by a reduced glomerular filtration rate was not significantly associated with any major ocular diseases, except for a marginally significant association with retinal vein occlusions [**37**].

Bilateral central retinal vein occlusion has happened before in a patient with chronic kidney disease leading to malignant hypertension and hyperviscosity [**38**]. In this case, however, the patient developed renal failure and malignant hypertension, a year after the onset of the ophthalmologic pathology.

Bilateral central retinal vein occlusion is a rare occurrence. The fact that the patient developed a drop in visual acuity in both eyes over a period of a few days pointed to the fact that some kind of systemic imbalance or disease must exist. In literature, cases of bilateral central retinal vein occlusion occurred in patients with chronic myeloid leukemia [**39**-**41**], acute myeloid leukemia [**42**], Waldenstrom's macroglobulinaemia [**43**-**45**], Hyperhomocysteinemia [**46**,**47**], multiple myeloma [**48**], Eisenmenger syndrome [**49**], occult colon cancer [**50**] and Sclerodermia [**51**]. These pathologies were excluded after analyzing the clinical, paraclinical and laboratory test results.

Dental procedures rarely induce ophthalmic complications including diplopia, strabismus, ptosis, and amaurosis fugax [**52**,**53**]. Injection of anesthetic solution into the oral cavity is the leading factor in the development of these complications. Besides this, tooth extraction is also charged for ocular complications [**52**-**55**]. More severe complications like permanent amaurosis, orbital cellulitis, orbital abscess, and endophthalmitis were reported in the literature [**54**]. Dental procedures under not only local anesthesia but also general anesthesia were shown to cause visual disturbances [**55**].

There is no consensus on the exact etiology of vascular ocular complications after dental procedure. Reported cases in the literature frequently developed after intraoral dental anesthesia but the mechanism is still not fully understood [**56**]. It is generally agreed that the local anesthetic solution reaches the orbit through vascular, neurological, or lymphatic network [**57**]. However, most cases involve retinal artery occlusion, through embolization or vasospasm, with no cases of retinal vein occlusion. 

There is the possibility that during the many dental interventions the patient received pro-coagulant substances for possible hemorrhaging. The toxic effect of some of the implanted materials could also be a cause for the vein occlusion, but the lack of knowledge about the toxic, irritant, and allergenic properties of dental materials prevents an exact conclusion on this matter.

The short-term prognosis for this case is moderate; the patient’s visual acuity has been stable for a while. The long-term prognosis, however, is uncertain. Due to general imbalance caused by the renal failure, the patient developed cardiac problems. Therefore, there is a risk of cerebrovascular and cardiovascular events. The patient could also develop diabetic retinopathy, because Tacrolimus, used as an immunosuppressant for the transplant, contributes to glucose intolerance [**58**-**62**] and chronic hepatitis C virus infection is also a risk factor for the development of diabetes [**63**,**64**].

**Financial Disclosures**

None of the other authors has any financial or proprietary interests to disclose.
